# Probiotics in the Prophylaxis of Premature Rupture of Membranes and Cervical Incompetence

**DOI:** 10.3390/nu16234230

**Published:** 2024-12-06

**Authors:** Constantin-Cristian Vaduva, Ana-Maria Petrescu, Laurentiu Mihai Dira, Dan Ruican, Razvan Cosmin Pana

**Affiliations:** 1 Department of Obstetrics and Gynecology, University of Medicine and Pharmacy, 200349 Craiova, Romania; laurentiu.dira@yahoo.com (L.M.D.); razvan.pana@umfcv.ro (R.C.P.); 2 Clinic of Obstetrics and Gynecology, Filantropia Clinical Hospital, 200143 Craiova, Romania; ruican.dan@hotmail.com; 3 Department of Obstetrics, Gynecology and IVF, HitMed Medical Center, 200130 Craiova, Romania; 4 Department of Mother and Child Medicine, Emergency County Hospital, 200642 Craiova, Romania

**Keywords:** probiotics, premature rupture of membranes (PROM), cervical incompetence (CI), vaginal microbiota, preterm birth

## Abstract

Premature rupture of membranes (PROM) and cervical incompetence (CI) are major contributors to preterm birth, a leading cause of neonatal morbidity and mortality. Background/Objectives: Disorders of the vaginal microbiota, such as bacterial vaginosis, have been associated with an increased risk of PROM, CI, and subsequent preterm birth. Probiotics, particularly Lactobacillus strains, have been proposed as a preventive strategy to restore and maintain a healthy vaginal microbiome. This review aims to summarize the latest evidence on the role of probiotics in the prevention of PROM and CI. Methods: A comprehensive review was conducted to evaluate the effectiveness of probiotic interventions in the prevention of PROM and CI, yielding 1809 records from 2005 to 2024. Seven relevant studies were selected by searching medical databases and focusing on studies that investigated the restoration of healthy vaginal flora, the reduction of pathogenic bacteria colonization, and the modulation of immune responses by probiotics. Results: The studies analyzed suggest that probiotics may help restore healthy vaginal flora, reduce pathogenic bacterial colonization, and modulate immune responses, thereby reducing the risk of membrane rupture and cervical insufficiency. Evidence from randomized controlled trials and observational studies shows that the use of probiotics is associated with a lower incidence of PROM and preterm birth, especially in high-risk groups. Conclusions: Probiotics emerge as a potential non-invasive and cost-effective strategy to improve pregnancy outcomes in women at risk of preterm birth due to PROM. According to our research, probiotic prophylaxis of cervical insufficiency has not yet been sufficiently investigated. Despite the promising findings, further research is needed to determine standardized probiotic formulations, optimal timing, and routes of administration. Personalized probiotic therapies may represent the future of preterm birth prevention as they offer targeted interventions based on individual microbiome composition.

## 1. Introduction

Premature rupture of membranes (PROM) and cervical incompetence (CI) are two critical conditions associated with preterm birth that contribute to significant neonatal morbidity and mortality. PROM is the rupture of the fetal membranes before the onset of labor, especially before 37 weeks gestation, and it is responsible for up to 30% of preterm births [1]. Cervical incompetence (CI), which is characterized by premature shortening or dilation of the cervix without uterine contractions, can also lead to preterm birth and increases the risk of adverse neonatal outcomes [2]. Both PROM and CI disrupt the protective barriers of pregnancy and lead to infections, premature births, and complications, such as neonatal sepsis, respiratory distress syndrome, and long-term developmental disorders [3,4,5].

In recent years, the role of the vaginal microbiome in the development of PROM and CI has become increasingly clear. The vaginal microbiota is primarily dominated by Lactobacillus species, particularly *L. crispatus* and *L. gasseri*, which play a protective role by maintaining a low pH environment that inhibits the growth of pathogenic bacteria [6,7]. Disruption of this balance, particularly with an over-representation of certain species, such as *Gardnerella vaginalis* and *Ureaplasma parvum*, known as bacterial vaginosis (BV), has been identified as a significant risk factor for PROM and preterm labor [8,9]. Studies suggest that BV and other infections associated with vaginal dysbiosis can lead to inflammation and weakening of the membrane, triggering an inflammatory cascade that can cause PROM or exacerbate CI [10,11,12].

Given the link between microbial dysbiosis and preterm birth, probiotics have shown promise as an intervention to reduce the risk of PROM and CI. Probiotics, particularly those containing Lactobacillus strains, have been shown to restore vaginal flora, reduce inflammation, and prevent the overgrowth of pathogenic bacteria that can lead to complications of preterm birth [13]. By promoting a balanced vaginal microbiome, probiotics may represent a non-invasive, cost-effective strategy to reduce the incidence of PROM and CI, particularly in high-risk groups [14].

This review examines the current literature on the efficacy of probiotics in the prevention of PROM and CI. It discusses the mechanisms by which probiotics affect the vaginal microbiome, the results of clinical trials, and the potential for integrating probiotics into routine antenatal care.

## 2. Materials and Methods

This review follows the PRISMA (Preferred Reporting Items for Systematic Reviews and Meta-Analyses) guidelines to ensure a comprehensive and structured synthesis of the available literature.

We searched four databases, Medline, PubMed, Cochrane Library, and Google Scholar, from 1 January 2005 to 10 January 2024. The language was restricted to English, and only full articles published in scientific journals were included in the search. Search terms included “probiotics’, “premature rupture of membranes’, “cervical incompetence”, “vaginal microbiota”, and “preterm birth prevention”.

Sources of gray literature—including conference abstracts, workshops, government reports, and studies with other designs—were excluded from this review. However, we examined the references in systematic reviews to find other relevant articles. Studies that examined generic probiotic products (e.g., probiotic foods or supplements) without specifying the dosage and microorganisms contained were not included. Secondary analyses of earlier studies were not included in this review, but they were screened for additional information not included in the main analyses and were cited in tables where appropriate. The inclusion criteria for this review were studies that investigated the role of probiotics in maintaining vaginal health during pregnancy, particularly in relation to the prevention of PROM and CI. Both observational studies and randomized controlled trials (RCTs) were included. This review focused on studies that specifically examined the effects of Lactobacillus species or other probiotics on pregnancy outcomes, such as the incidence of PROM, preterm labor, and infection-related complications.

All identified studies were screened for the year, citation, title, authors, and abstract. Duplicates were identified through manual screening by one researcher and subsequently removed. Two authors independently reviewed the titles and abstracts of all unduplicated papers and excluded those that were not relevant to the topic.

The same two authors independently reviewed the full text of the papers that passed the initial screening and selected those to be included in the review.

## 3. Results

**Scheme 1:**
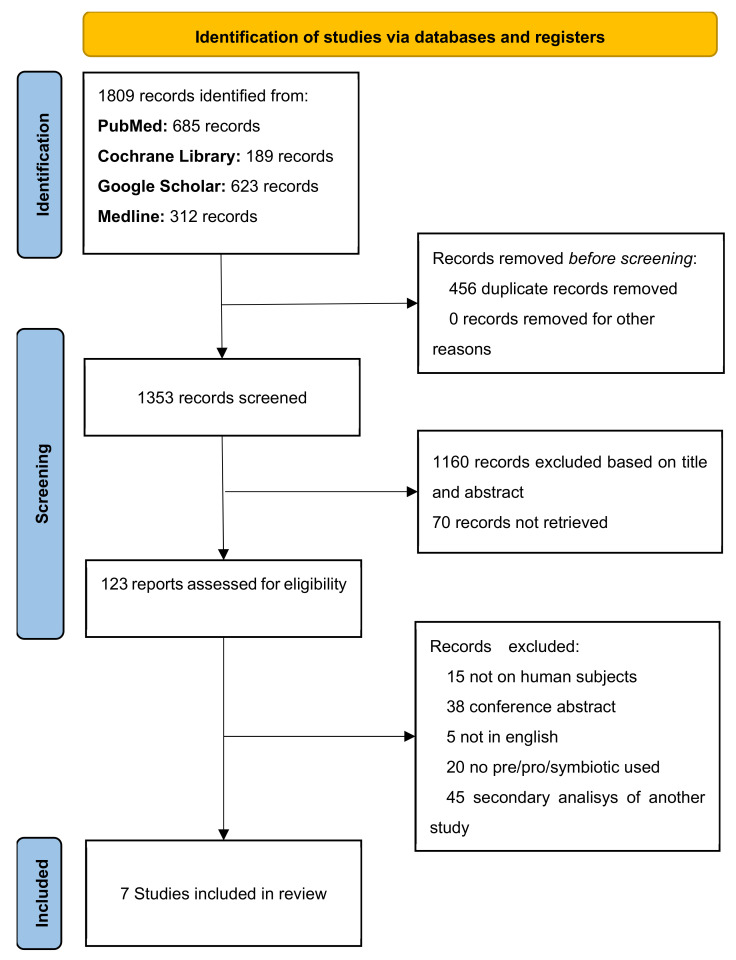
PRISMA diagram of the eligible studies included in the review.

We identified a total of 1809 studies from four databases: PubMed, Cochrane Library, Google Scholar, and Medline. We removed 456 duplicates; thus, 1353 records were screened for title and abstract review, and 123 reports were eligible for full-text review. Finally, we excluded 116 papers because they did not meet the inclusion criteria, whether they were not in humans (*n* = 15), conference abstracts (*n* = 38), not in English (*n* = 5), did not use a probiotic (*n* = 5), or secondary analyses of another study (*n* = 45). The flowchart for study selection is shown in Scheme 1.

Table 1 below summarizes the reports investigating the use of probiotics for PROM prophylaxis. It includes details regarding the type of probiotic strains used, the methods of administration, the number of patients, including the number of patients who responded to probiotics, the mean gestational age (MGA) at inclusion, and the MGA at delivery for both the probiotic and control groups. These studies shed light on the potential efficacy of probiotics in prolonging pregnancy.

Although numerous studies have been carried out on the effect of probiotics on premature rupture of the membranes and their effect was clearly positive, the type of probiotic strain used in the studies varied. The method of administration also varies between studies.

Petrova et al. studied *Lactobacillus rhamnosus* GG in a cohort of 120 pregnant women and found a significant improvement in gestational age in those who received probiotics. In the group that responded positively to the probiotic, the mean gestational age at delivery was 37.0 weeks, compared to 35.0 weeks in the group that did not receive it. Oral administration of *Lactobacillus rhamnosus* GG has been shown to be effective in improving the health of the vaginal microbiota and reducing the risk of preterm birth by improving the microbial balance. The study emphasizes the importance of oral probiotic strains that support the natural presence of *Lactobacillus* in the vaginal environment [15].

The EFFPRO study by Gille et al. contributed to the understanding of the oral administration of probiotics by using a mixture of Lactobacillus strains in 198 women. The results were promising: 130 women showed positive responses and an increased mean gestational age at delivery of 36.4 weeks. In contrast, the mean gestational age in the control group was 34.2 weeks, suggesting that oral lactobacilli may support vaginal health and potentially reduce the risk of preterm birth and other complications. The study supports the idea that oral administration of probiotics may be beneficial and may be more accessible to broad populations than topical (vaginal) administration [16].

In a Cochrane review, Othman et al. investigated various strains of probiotics in several randomized studies. The study of 238 participants aimed to understand the potential of probiotics to prevent preterm birth by modulating the vaginal microbiome and reducing infections, a common cause of preterm birth. Women who responded to probiotic treatment had a mean gestational age of 35.5 weeks, compared to 33.5 weeks in the control group. The comprehensive review emphasizes that strain diversity and context—such as high-risk cases—play a crucial role in the efficacy of probiotics and demonstrate the potential of different probiotic strains in different populations [17].

In the study by Samanta et al., the combination of *Lactobacillus* GG and *Bifidobacterium breve* showed similar effects, with 112 out of 180 participants reacting positively. The study showed an increase in the average gestational age in the probiotic users to 36.3 weeks compared to 34.1 weeks in the group without probiotics. The study highlights the effectiveness of a combined approach, especially when there is a risk of PROM and bacterial vaginosis (BV), which are known to cause preterm labor. Samanta et al. showed that Lactobacillus and Bifidobacterium strains, especially when administered orally, contribute significantly to reducing inflammatory responses, supporting a balanced microbiota [18].

Benor et al. investigated the vaginal administration of *Bifidobacterium longum* and *Lactobacillus acidophilus* in 210 patients, 135 of whom reacted positively. The probiotic patients had a mean gestational age of 36.7 weeks, compared with 33.9 weeks in the control group. The study shows that vaginal administration can be particularly beneficial when local dysbiosis is a problem, such as in women with BV or high susceptibility to infection. Vaginal administration may deliver probiotics more directly to the target site, which could be beneficial for patients with recurrent vaginal infections or at high risk of microbial imbalance [19].

The study by Krauss-Silva et al. also investigated the role of probiotics in the prevention of preterm birth, focusing specifically on *Lactobacillus rhamnosus* GR-1 and *Lactobacillus reuteri* RC-14. This randomized, controlled trial, conducted with a cohort of 220 pregnant women, investigated the potential of probiotics to reduce the incidence of spontaneous preterm birth in women at increased risk of bacterial vaginosis. The results showed a significant decrease in BV-related preterm births in the women who received probiotics, with an increase in gestational age in this group compared to the control group. These results support the theory that local probiotic interventions can restore the health of the vaginal microbiome and reduce the risk of preterm birth associated with infections and microbial imbalances [20].

In our search, we were unable to find any direct studies that investigated the use of probiotics for the prophylaxis of cervical insufficiency. Although we included cervical insufficiency in our search criteria and title, there are no studies in the current literature that specifically address the role of probiotics in the prevention or treatment of this condition. This absence indicates a gap in existing knowledge and suggests that the potential effects of probiotics on cervical integrity remain unexplored. There are reports on the use of probiotics in pregnant women who have undergone cervical cerclage, with no statistically significant differences between the probiotic group and the control group in terms of preterm delivery [21]. However, the group size was not sufficient to detect changes in the incidence of preterm birth, but probiotics were associated with a lower likelihood of premature rupture of the bladder.

**Scheme 2:**
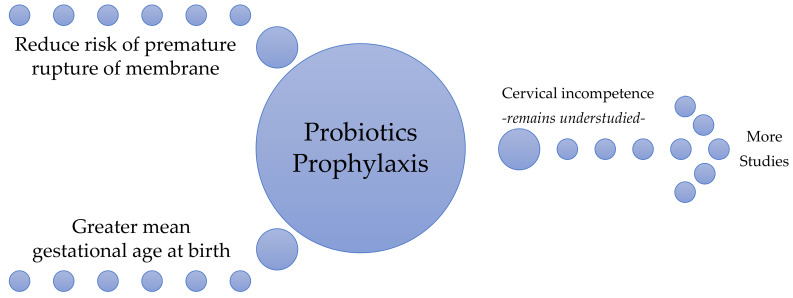
Summary of results. Probiotic prophylaxis may reduce the risk of premature rupture of membranes and improve the mean gestational age at delivery. The effects on cervical incompetence have not yet been sufficiently researched.

Overall (Scheme 2), these studies suggest that probiotics, particularly when administered as mixed strains and through tailored delivery methods, show promise in improving gestational age and reducing the risk of PROM and preterm birth. Oral and vaginal routes of administration each show unique benefits, with vaginal administration potentially providing a more direct benefit to microbial balance in women at high risk of preterm birth complications.

### Meta-Analyses

A meta-analysis by Abavisani et al. emphasized the role of probiotics in reducing the risk of infection [22]. It included more than 3571 participants and concluded that women who received probiotics had a significantly higher cure rate for bacterial vaginosis and vulvovaginal candidiasis compared to the control group. The review also found that probiotics were well-tolerated and did not increase the risk of adverse pregnancy outcomes. Although the study did not include pregnant women, the positive effect of probiotics as an adjunct treatment to antibiotics is emphasized.

A large-scale meta-analysis published in the British Journal of Nutrition in 2020 summarized data from 4356 patients from studies using predominantly oral mixed strains of Lactobacillus and Bifidobacterium and found a mean gestational age of 37.5 weeks in probiotic users compared to 34.9 weeks in non-users [23]. The large sample size and mixed-strain approach resulted in a notable increase in gestational age, suggesting that mixed strains are effective at the population level and that oral administration may be accessible and beneficial for various pregnancy risks.

Jarde et al. conducted a systematic review and a meta-analysis to evaluate the effects of probiotics and prebiotics on pregnancy outcomes. The study included data from pregnant women who received probiotics or prebiotics compared to a placebo or no treatment. The results showed no significant effects on outcomes, such as pre-eclampsia, preterm birth, or birth weight [24]. Pérez-Castillo et al. conducted a systematic review and meta-analysis to assess the reporting of perinatal outcomes in RCTs of probiotic interventions. The study assessed the quality and consistency of reporting of perinatal outcomes, such as preterm birth, birth weight, and neonatal complications in different RCTs. The results showed considerable heterogeneity and inconsistency in the reporting of outcomes, which made it difficult to effectively compare and synthesize results. The authors emphasized the need for standardized reporting guidelines to improve the reliability and comparability of future research on probiotics and perinatal health outcomes [25]. However, these reviews are problematic because most of the included studies did not examine preterm birth as a primary outcome. In addition, the authors included studies in which the probiotic was administered from 36 weeks gestation, which would not affect preterm birth, as 7 days later is considered full term.

## 4. Discussion

The role of probiotics in the prevention of premature rupture of membranes and cervical insufficiency has received increasing attention due to the crucial role that the vaginal microbiome plays in maintaining pregnancy. A healthy balance of the microbiota in the vagina, which is primarily dominated by Lactobacillus species, is crucial in preventing bacterial overgrowth that could lead to infection, inflammation, and, ultimately, premature rupture of the membranes or cervical insufficiency.

### 4.1. Mechanisms of Action

An important mechanism by which probiotics prevent PROM and CI is their ability to restore and maintain the vaginal microbiota. The vaginal microbiota changes dynamically during pregnancy, and a healthy microbiome dominated by Lactobacillus species is considered protective against infections that can trigger premature birth [10]. Lactobacillus species, especially *L. crispatus* and *L. gasseri*, produce lactic acid, which lowers the vaginal pH to values below 4.5, creating an unfavorable environment for pathogenic bacteria, such as *Gardnerella vaginalis* and *Ureaplasma parvum*, both of which are associated with bacterial vaginosis and PROM [26].

Probiotics also exert an anti-inflammatory effect by down-regulating inflammatory pathways that can compromise cervical integrity. For example, *Lactobacillus rhamnosus* GR1 and *Lactobacillus reuteri* RC14 have been shown to increase the production of anti-inflammatory cytokines, such as IL-10, and reduce the levels of pro-inflammatory cytokines, such as TNF-α [27,28,29]. By modulating the immune response, these probiotics help prevent the inflammatory cascade, which can lead to premature cervical shortening or weakening, which is characteristic of cervical incompetence.

In addition, the production of antimicrobial substances, such as hydrogen peroxide, bacteriocins, and biosurfactants, by Lactobacillus species inhibits the growth of pathogenic bacteria and thus supports a healthy vaginal microbiota. Studies have shown that taking probiotics not only reduces the incidence of bacterial vaginosis but also increases the colonization of the vaginal tract with beneficial bacteria, which contributes to a more stable and protective microbiome [30,31].

### 4.2. Probiotics in High-Risk Populations

A recurring theme in the literature is the benefit of probiotics in high-risk groups, particularly women with a history of preterm birth, bacterial vaginosis, or other pregnancy complications. Women with a history of preterm birth have an increased risk of recurrent preterm birth in subsequent pregnancies, and this risk is exacerbated by vaginal dysbiosis [32]. Several studies have shown that the administration of probiotics in early pregnancy, especially in women with a history of vaginal infections, can significantly reduce the incidence of preterm birth and cervical incompetence [33,34,35]. There are four randomized controlled articles on probiotics for prolonging pregnancy even after PROM [36,37,38,39]. However, we have focused on the prophylaxis of PROM.

In a Cochrane review, pregnant women with a history of preterm birth received probiotics containing *L. rhamnosus and L. reuteri* from early pregnancy to delivery. The results showed a significant reduction in the incidence of PROM and preterm birth in the probiotic group compared to the control group [17]. The study also found improvements in the composition of the vaginal microbiota, with a significant increase in Lactobacillus dominance, which is consistent with the findings of other authors [10,17].

Chronic inflammation during pregnancy is increasingly recognized as a critical factor that has a negative impact on obstetrics, including premature birth and cervical insufficiency [10]. The vaginal microbiome plays a central role in the modulation of inflammatory reactions within the reproductive tract. A microbial shift promotes the production of pro-inflammatory cytokines and chemokines and triggers a chronic inflammatory state. Such inflammation can weaken the cervical extracellular matrix and fetal membranes, increasing the risk of premature cervical dilatation and rupture of the membranes [40]. Understanding the interplay between chronic inflammation and the vaginal microbiome is crucial for the development of therapeutic interventions, such as the use of probiotics, to restore microbial balance and prevent inflammation-related pregnancy complications. The vaginal microbiome of women with endometriosis is often altered, with a decrease in beneficial Lactobacillus species and an increase in pathogenic bacteria, such as *Gardnerella vaginalis* and *Atopobium vaginae*. Chronic inflammation and fibrosis associated with endometriosis can also affect the function of the placenta, which can lead to pregnancy loss [41]. Surgical treatment before conception can help to reduce some of these risks, but close monitoring during pregnancy remains essential for women with severe endometriosis [42,43]. Studies suggest that interventions aimed at restoring the balance of the vaginal microbiome, such as probiotics, can reduce inflammation and improve pregnancy outcomes in women with endometriosis [44,45].

### 4.3. Challenges and Limitations

Despite the promising results of many studies, there are challenges and limitations related to the use of probiotics in the prevention of PROM and CI. In our search, we found that prophylaxis with probiotics in PROM and especially in CI has not yet been sufficiently researched. Because PROM and CI are closely related to preterm labor and they are important risk factors for preterm labor, we decided that our study needs to investigate how these probiotics affect preterm labor. Another major challenge is the heterogeneity of the probiotic formulations used in the different studies. Different Lactobacillus strains have different properties, and not all strains are equally effective in colonizing the vaginal tract or preventing dysbiosis [46]. For example, *L. rhamnosus* GR1 and *L. reuteri* RC14 have been shown to be very effective in several studies, while other strains, such as *L. acidophilus*, have not shown the same degree of efficacy [47].

Another limitation is the different timing and route of administration of probiotics. Some studies used oral probiotics, while others focused on vaginal administration. While oral probiotics have the advantage of being non-invasive and easy to administer, they may not always result in adequate colonization of the vaginal tract, especially in women with severe dysbiosis [48]. Vaginal probiotics, on the other hand, can lead to more direct and faster colonization of the vaginal microbiota, but they are more difficult to administer, and they can be associated with compliance problems [49].

Furthermore, the optimal time for the administration of probiotics is still unclear. Some studies suggest that probiotics should be administered early in pregnancy to establish and maintain a healthy vaginal microbiome throughout pregnancy, while other studies suggest that probiotics are most effective when administered in the second or third trimester, especially in women with a history of preterm labor or bacterial vaginosis [50]. Future research should aim to establish standardized guidelines for the use of probiotics, including the most effective strains, dosages and timing of administration.

### 4.4. Future Directions

Although the current evidence suggests that probiotics may reduce the risk of PROM and cervical failure, further research is needed to confirm these findings and to fill the remaining knowledge gaps, particularly in relation to the topic of probiotics and cervical failure.

In addition, the role of other microbial species in the vaginal microbiota besides Lactobacillus needs to be investigated. While most studies have focused on the Lactobacillus-dominated microbiota, new research suggests that other microbial species, such as Bifidobacterium and Streptococcus, may also play a role in preventing infection and maintaining cervical integrity.

New research suggests that maternal infections associated with vaginal dysbiosis, particularly in the first trimester, may be linked to congenital abnormalities in the fetus, such as neural tube defects, cardiac malformations, and even fetal ovarian cysts [51,52,53]. This association highlights the importance of monitoring and managing the vaginal microbiome during pregnancy to reduce inflammation and support healthy fetal development.

Another area of growing interest is the potential for personalized probiotic therapies based on individual microbiome profiles. It is known that the vaginal microbiota varies greatly from person to person and that certain factors, such as genetics, diet, and previous antibiotic use, can influence the microbial composition [32]. Therefore, a one-size-fits-all solution for the administration of probiotics may not be effective for all women. Personalized probiotic therapies tailored to the individual composition of a woman’s microbiome may offer a more targeted and effective approach to the prevention of PROM and CI [54].

Advances in microbiome sequencing technology have made it possible to identify women at risk of PROM or cervical insufficiency based on the composition of their vaginal microbiota. Studies have shown that women with a more diverse vaginal microbiota, particularly with an excess of anaerobic bacteria, have an increased risk of preterm birth [55]. If women with dysbiosis are detected early in pregnancy, healthcare providers could offer personalized probiotic treatments to restore a healthy balance of Lactobacillus species and reduce the risk of complications.

## 5. Conclusions

In summary, probiotics represent a promising and potentially non-invasive approach to the prevention of premature rupture of membranes and cervical incompetence. By restoring and maintaining a healthy vaginal microbiome, probiotics may reduce the risk of bacterial vaginosis, inflammation, and subsequent complications leading to preterm birth. While more research is needed to establish standardized guidelines for the use of probiotics in pregnancy, the existing literature supports their use in high-risk groups. The future of probiotic therapy may lie in personalized medicine, with treatments tailored to an individual’s unique microbiome profile, providing a targeted and effective approach to improving pregnancy outcomes.

## Figures and Tables

**Table 1:** Characteristics of the included studies. MGA—mean gestation age.

Study	Type of Probiotic	Administration Method	No. of Patients	Responded to Probiotics (*n*)	MGA Inclusion (weeks)	MGA at Birth with Probiotics (weeks)	MGA at Birth without Probiotics (weeks)
**Petrova et al. (2015)**	*Lactobacillus rhamnosus* GG	Oral	120	80	34.5	37	35
**Gille et al. (EFFPRO) (2016)**	Oral lactobacilli strains	Oral	198	130	33.8	36.4	34.2
**Othman et al. (Cochrane Review) (2007)**	Various strains in randomized trials	Various	238	150	34	35.5	33.5
**Samanta et al. (2022)**	*Lactobacillus* GG and *Bifidobacterium breve*	Oral	180	112	34.2	36.3	34.1
**Benor et al. (2022)**	*Bifidobacterium longum* and *Lactobacillus acidophilus*	Vaginal	210	135	33.8	36.7	33.9
**PiP Study (New Zealand) (2016)**	*Lactobacillus rhamnosus* HN001	Oral	300	190	34.7	37	35.2
**Krauss-Silva et al. (2011)**	*Lactobacillus rhamnosus* GR-1 and *Lactobacillus reuteri* RC-14	Oral	220	145	33.9	36.2	34.4

## Data Availability

The original contributions presented in this study are included in the article. Further inquiries can be directed to the corresponding authors.
